# Molecular and Clinical Characterization of a Novel Nonsense Variant in Exon 1 of the *UPF3B* Gene Found in a Large Spanish Basque Family (MRX82)

**DOI:** 10.3389/fgene.2019.01074

**Published:** 2019-10-31

**Authors:** María Isabel Tejada, Olatz Villate, Nekane Ibarluzea, Ana Belén de la Hoz, Cristina Martínez-Bouzas, Elena Beristain, Francisco Martínez, Michael J. Friez, Beatriz Sobrino, Francisco Barros

**Affiliations:** ^1^Genetics Service, Cruces University Hospital, Osakidetza Basque Health Service, Barakaldo, Spain; ^2^Biocruces Bizkaia Health Research Institute, Barakaldo, Spain; ^3^Spanish Consortium for Research on Rare Diseases (CIBERER), Valencia, Spain; ^4^Molecular Genetics Laboratory, Araba University Hospital, Osakidetza Basque Health Service, Vitoria-Gasteiz, Spain; ^5^Servicio de Genética, Hospital Universitario y Politécnico La Fe, Valencia, Spain; ^6^Greenwood Genetic Center, Greenwood, SC, United States; ^7^Fundación Pública Galega de Medicina Xenómica, Grupo de Medicina Xenómica (USC), Santiago de Compostela, Spain

**Keywords:** *UPF3B*, next generation sequencing, intellectual disability, non-syndromic X linked intellectual disability, autism spectrum disorder

## Abstract

X-linked intellectual disability (XLID) is known to explain up to 10% of the intellectual disability in males. A large number of families in which intellectual disability is the only clinically consistent manifestation have been described. While linkage analysis and candidate gene testing were the initial approaches to find genes and variants, next generation sequencing (NGS) has accelerated the discovery of more and more XLID genes. Using NGS, we resolved the genetic cause of MRX82 (OMIM number 300518), a large Spanish Basque family with five affected males with intellectual disability and a wide phenotypic variability among them despite having the same pathogenic variant. Although the previous linkage study had mapped the locus to an interval of 7.6Mb in Xq24–Xq25 of the X chromosome, this region contained too many candidate genes to be analysed using conventional approaches. NGS revealed a novel nonsense variant: c.118C > T; p.Gln40* in *UPF3B*, a gene previously implicated in XLID that encodes a protein involved in nonsense-mediated mRNA decay (NMD). Further molecular studies showed that the mRNA transcript was not completely degraded by NMD. However, UPF3B protein was not detected by conventional Western Blot analysis at least downstream of the 40 residue demonstrating that the phenotype could be due to the loss of functional protein. This is the first report of a premature termination codon before the three functional domains of the UPF3B protein and these results directly implicate the absence of these domains with XLID, autism and some dysmorphic features.

## Background

Intellectual Disability (ID) and Autism Spectrum Disorder (ASD) are serious medical and social problems in developed countries where ID prevalence has been estimated at 1% (Maulik et al., 2011). ID can occur in many occasions in combination with ASD (Vissers et al., 2015), and both conditions are highly heterogeneous with a strong genetic component (Bailey et al., 1995).

The X chromosome is of particular interest because it is known to harbor more than 10% of the ID genes (Chiurazzi and Pirozzi, 2016). Since the discovery of the Fragile X locus, a great effort has been done to identify the cause of X-linked Intellectual Disability (XLID). While linkage analyses and candidate gene testing were the initial approaches, Next Generation Sequencing (NGS) has now accelerated the discovery of more and more XLID genes. In this way, more than 141 XLID genes have been identified to date (Neri et al., 2018), a large number of them being responsible for non-syndromic XLID (NS-XLID), in which ID is the only clinically consistent manifestation.

We report the resolution of MRX82 (OMIM number 300518), a large Spanish Basque family with five affected males with a high phenotypic variability (Martínez et al., 2004). Although a previous linkage study mapped the locus to an interval of 7.6Mb in Xq24–Xq25, this region contained too many candidate genes to be analyzed using conventional approaches. However, NGS revealed a novel nonsense variant: c.118C > T; p.Gln40* in *UPF3B*, a gene previously implicated in XLID (MIM 300298).

*UPF3B* encodes the Regulator of nonsense transcripts 3B (REN3B) protein initially identified as a component of an exon–junction complex that promotes nonsense-mediated mRNA decay (NMD). Some authors have studied the functions of this gene (Kunz et al., 2006) and their implication in ID. Tarpey et al. (2007) described for the first time hemizygous variants in the *UPF3B* gene in affected males of 4 unrelated families. The described phenotype was variable, including mild to severe ID and autistic features. Three of these families had originally been diagnosed as having Opitz-Kaveggia and Lujan-Fryns syndromes, and the fourth had NS-XLID. Since then, more and more patients with ID have been studied by NGS and more variants have been reported in the *UPF3B* gene. To date, 21 variants (17 pathogenic) have been identified in *UPF3B* according to the Human Gene Mutation Database (HGMD)^1^ and 6 other pathogenic in ClinVar^2^, 4 of which overlap with the HGMD. However, clinical characteristics of patients in these massive sequencing studies were not described.

Due to the limited number of published cases with extensive phenotypic description, the aim of this report is to describe and characterize a novel variation in *UPF3B* and provide further insights into the wide clinical spectrum produced by the absence of UPF3B protein.

### Case Presentation

**Figure 1:**
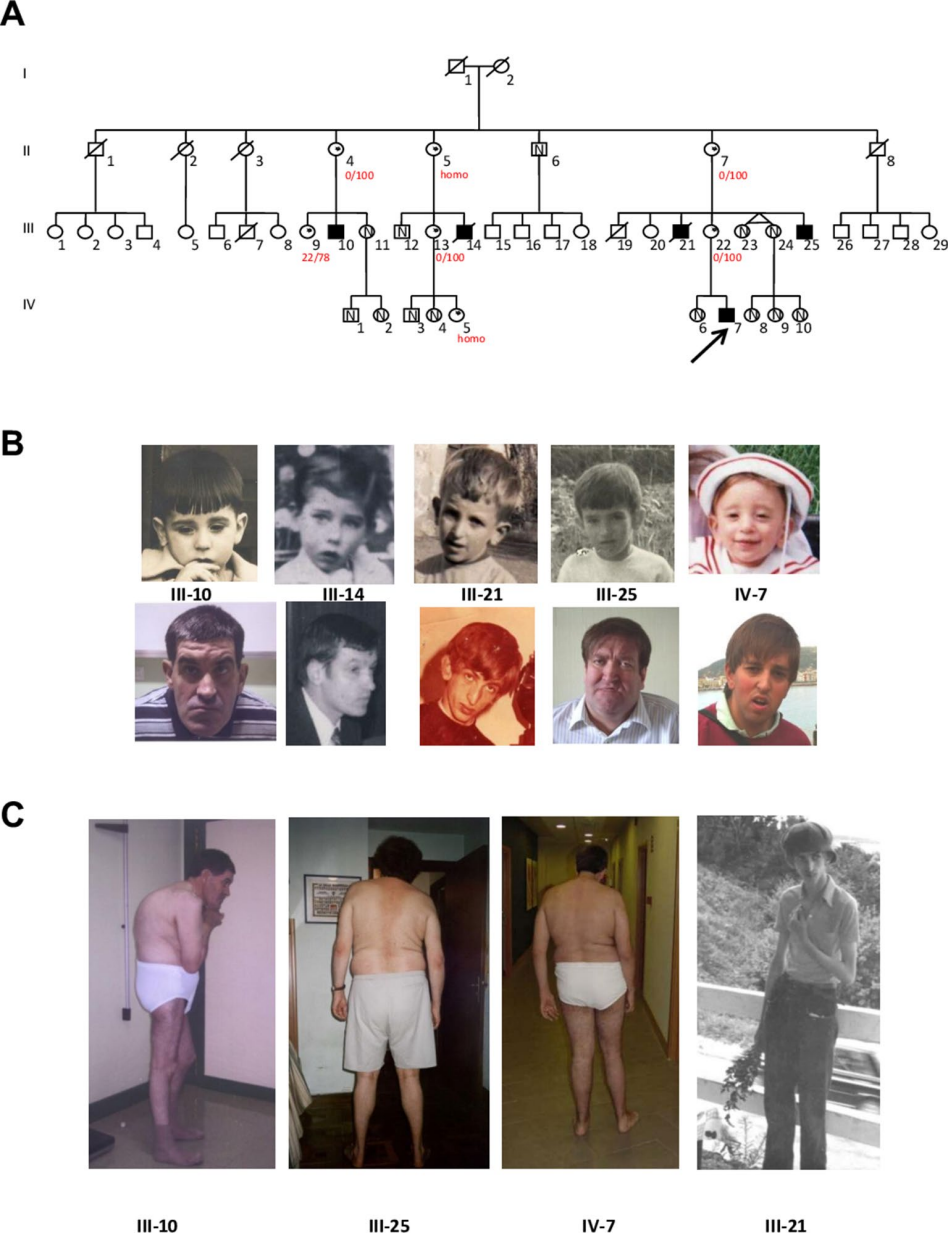
**(A)** Updated Pedigree of the family showing the five affected males (blackened squares) related through their clinically normal carrier mothers (dots within the circles); N indicates the presence of the normal (wild type) allele in the studied individuals. The proband is indicated by the arrow. Numbers in red represent X Inactivation (homo: homozygous for the AR repeat). **(B)** Facial features of the five affected members in childhood and adulthood. **(C)** Scoliosis, kyphosis and marfanoid habitus in four affected males.

The pedigree of this Spanish Basque family is shown in **Figure 1A**. The five affected males are maternally related through four normal obligate carriers. They have mild to profound ID and two of them have Autism Spectrum Disorder (ASD) according to Autism Diagnostic Observation Schedule (ADOS). Clinical description of the family was published previously (Martínez et al., 2004) and it was classified as having NS-XLID because they showed a wide phenotypic variability among them. Following the identification of the variant, mothers of the affected males provided photographs (**Figures 1B, C**) and more clinical information. However clinical re-examination was not performed. **Table 1** summarizes the specific morphological and neurological signs present or not, and provides comparison with other published cases. As it can be seen, there is a remarkable clinical variability. Interestingly, one of the patients presented with a clear marfanoid habitus (**Figure 1C**) while the others did not. In relation to skeletal abnormalities, four of them presented with scoliosis of variable severity and one of them kyphosis (**Figure 1C**). The proband (IV-7) experienced seizures from 6 to 12 months of age after which no more seizures occurred. III-21 and III-25 started having epileptic seizures at 20 and 39 years respectively which responded well to treatment. Finally, the wide range of IQ values is striking, ranging from mild to profound.

**Table 1:** Summary of main clinical features in the 5 affected males from the family and comparison with other patients described in the literature.

Clinical findings	Affected males from this family					Other reports		
	IV-7 (Proband)	III-10	III-14	III-21	III-25	Tarpey et al. (2007)*	Laumonnier et al. (2010)*	Lynch et al. (2012)
Age at examination	18 years	51 years	Died at 45 years of an embolism	Died at 22 years of a heart attack	46 years			
Height >97th	Yes	No	Yes	Yes	Yes		No	No
Weight >97th	Yes	No	No	No	Yes		No	No
Head circumference >97th	Yes	No	No	Yes	Yes		No	1/2
**Facial features**								
Long face	Yes	Yes	No	Yes	Yes	Yes		2/2
Broad forehead	Yes	No	No	Yes	No	Yes	Yes	1/2
Deep set eyes	Yes	Yes	Yes	No	Yes			1/2
High nasal bridge	Yes	No	Yes	Yes	Yes	Yes		2/2
Long narrow nose	Yes	Yes	Yes	Yes	Yes			1/2
Short philtrum	Yes	Yes	Yes	Yes	Yes			2/2
High arched palate	Yes	No	Not examined	Not examined	Yes	Yes		
**Musculoskeletal features**								
Hypotonia	Yes	No	No	No	No		Yes	1/2
Slender build/poor musculature	No	No	No	Yes	No	Yes		
Scoliosis	Yes	Yes	?	Yes	Yes		Yes	
Kyphosis	No	Yes	No	No	No		Yes	
Marfanoid-like features	No	No	No	Yes	No	Yes	Yes	
Long, thin hyperextensible fingers and toes	Yes	No	Yes	Yes	Yes		Yes	
**Neurodevelopment**								
Degree of Intellectual Disability	Moderate: IQ < 50	Profound: IQ = 20,5	Mild: IQ < 70	Moderate: IQ < 50	Mild: IQ = 67	Mild to severe	Borderline to severe	Mild and moderate
Seizures	Yes	No	No	Yes	Yes		Yes	No
Speech impairment	Absent speech	Absent speech	Yes	Absent speech	Yes			½
Stereotypies	Yes	No	Yes	No	No			2/2
ASD (using ADOS)	Yes	No	No	Yes	No	Yes		2/2
Behavioral problems	Yes	No	No	No	Yes	Yes		2/2
Brain CT scan or MRI	Normal	Normal	Not examined	Not examined	Normal			Normal

In the articles by Tarpey et al. (2007) and Laumonnier et al. (2010) (*) “Yes” means that there are some patients with the clinical signs but not all. In Lynch et al. (2012) there are only two patients described.

All carrier women have normal intelligence and no dysmorphic features. Clinical information and blood samples were obtained from every participating member of the family or their legal representatives (mothers) in case of affected males after signing the written informed consent. The study was approved by the research ethics committee of Cruces University Hospital, Barakaldo, Spain. Written informed consent was also signed for the publication of photographs of affected individuals.

## Materials and Methods

The following tests were performed in the proband before NGS and yielded normal results: karyotype, Fragile X syndrome, MLPA assay with subtelomeric probes and array CGH (180k). X-linkage study mapped the locus to the long arm of chromosome X, in an interval of 7.6Mb in Xq24–Xq25 (Martínez et al., 2004).

### Next Generation Sequencing and Confirmation of the Variant by Sanger Sequencing

Genomic DNA was extracted from peripheral blood. The sequencing of the complete exome of two affected males (III-25 and IV-7) was performed using the equipment SOLID4 (Life Technologies) from 3 µgs of DNA. The whole procedure was carried out in the genomic sequencing platform of the Galician Public Foundation of Genomics Medicine in Santiago de Compostela, Spain.

Enrichment was done using the Agilent Technologies SureSelect All Exon v1 Kit. Prior to enrichment, the genomic libraries of the samples were constructed following the protocol SureSelect Target Enrichment System from the Applied Biosystems SOLiD System, Protocol v 1.7. The enriched Fragmented Libraries were sequenced following the Paired-end protocol 50 + 35bp sequencing.

Quality control, mapping, pairing and variant calling were made using Lifescope (Life Technologies). For the annotation, ANNOVAR was used and data were filtered using different databases (dbSNP, 1000 Genomes, HMDB). The results were checked by conventional Sanger sequencing using the BigDye Terminator v 3.1 Cycle Sequencing Kit.

Independently, the variant was also sequenced in IV-7 DNA at the Greenwood Genetic Center (USA). Briefly: A standard Next Generation Sequencing protocol for Whole Genome Sequencing (WGS) utilizing an Illumina NovaSeq 6000 generated raw data for secondary analysis. An in-house pipeline aligned the raw FASTQ files which produced an alignment with an average WGS coverage of 54X with 99.86% of nucleotides having greater than 10X coverage. Exonic and flanking variants were filtered using Alissa Interpret software (Agilent, USA) with the subsequent short list of X-linked variants being manually reviewed and curated.

### X Inactivation

The X-chromosome inactivation (XCI) analysis was performed on peripheral blood of the seven carrier females following the protocol described previously (Allen et al., 1992). Briefly, genomic DNA was digested using the HhaI (Takara Bio Inc., Japan) and HpaII (Takara Bio Inc., Japan) restriction enzymes and the 5′ UTR repetitive region of the Androgen Receptor (AR) gene was then amplified.

### RT-PCR and cDNA Analysis

cDNA was obtained using Superscript RT II enzyme (Invitrogen, Carlsbad, CA, USA) from 500 ng of total RNA extracted from blood in a volume of 20 µl. Partial cDNA of *UPF3B* gene was then amplified and sequenced.

### PBMCs Isolation and Western Blot Analysis

Peripheral blood mononuclear cells (PBMCs) were isolated from 10 ml of blood using LymphoprepTM (Stem Cell Technologies). PBMCs were lysed and frozen at −80 °C.

Immunoblot analysis was performed on equivalent amounts of protein extracts from the proband (IV-7), his mother (III-22), the affected uncle (III-25) and an unrelated normal control. Total protein was extracted and resolved by 6–12% sodium dodecyl sulphate-polyacrylamide gel electrophoresis, transferred to a nitrocellulose membrane and immunoblotted with the specific antibodies for the protein of interest. Rabbit anti-UPF3B antibody, Affinity Purified (A303-688A-T, Bethyl Laboratories) and mouse anti-GAPDH (sc-32233, Santa Cruz) were used as primary antibodies. After washing, secondary antibodies (IRDye 800CW goat anti-rabbit 926-32211, IRDye 680RD goat anti-mouse 926-68070; Li-Cor Biosciences, dilution 1/15,000) were incubated for 1 h and images were acquired with an Odyssey Clx imager after careful washing of the membranes.

## Results

### Identification of the Variant

**Figure 2:**
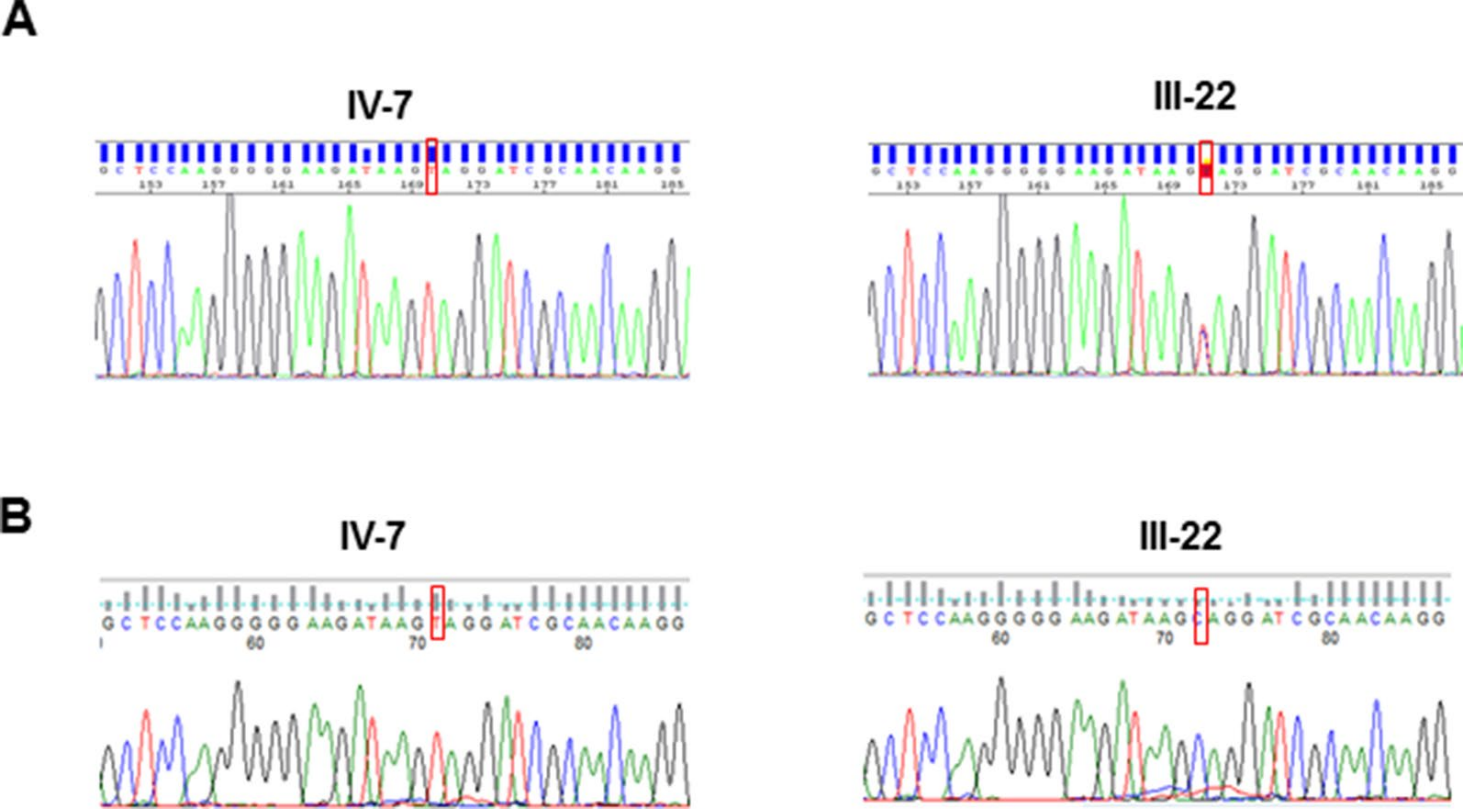
Sanger sequences of the *UPF3B* variant identified in this study: **(A)** in DNA from the proband (IV-7) and his mother (III-22); **(B)** in cDNA from the proband (IV-7), expressing the transcript with the pathogenic variant and his mother (III-22), who expresses only the normal transcript.

Taking advantage of the previous linkage analysis which located the putative variant in an interval of 7.6Mb in Xq24–Xq25, the bioinformatic analysis and variant filtering firstly focused on this region and identified a nonsense variant c.118C > T (p.Gln40*) in the *UPF3B* gene (**Figure 2A-1**). This variant was not present in any of the population databases checked (Exome Aggregation Consortium, 1000 Genomes, and Exome Variant Server) nor has it been reported in the literature. By Sanger sequencing, the presence of this variant was validated in the proband and studied in the other two living affected males, in seven carrier females (**Figure 2A-2**) and in 13 asymptomatic members of the family, resulting in a complete co-segregation within the family (**Figure 1A**).

### X Inactivation and cDNA Analysis

XCI profile on five female carriers was determined and revealed a complete skewed X-inactivation (0:100) in four of them (II-4, II-7, III-13 and III-22) and 22:78 in III-9. Two other females were homozygous (not informative) for the AR repeat (II-5 and IV-5). cDNA sequence on the proband's mother showed that only the wild type allele is expressed (**Figure 2B-2**). Furthermore, cDNA sequence analyses of the proband ruled out any other possible alteration at the RNA sequence level (**Figure 2B-1**).

### Western Blot Analysis

**Figure 3:**
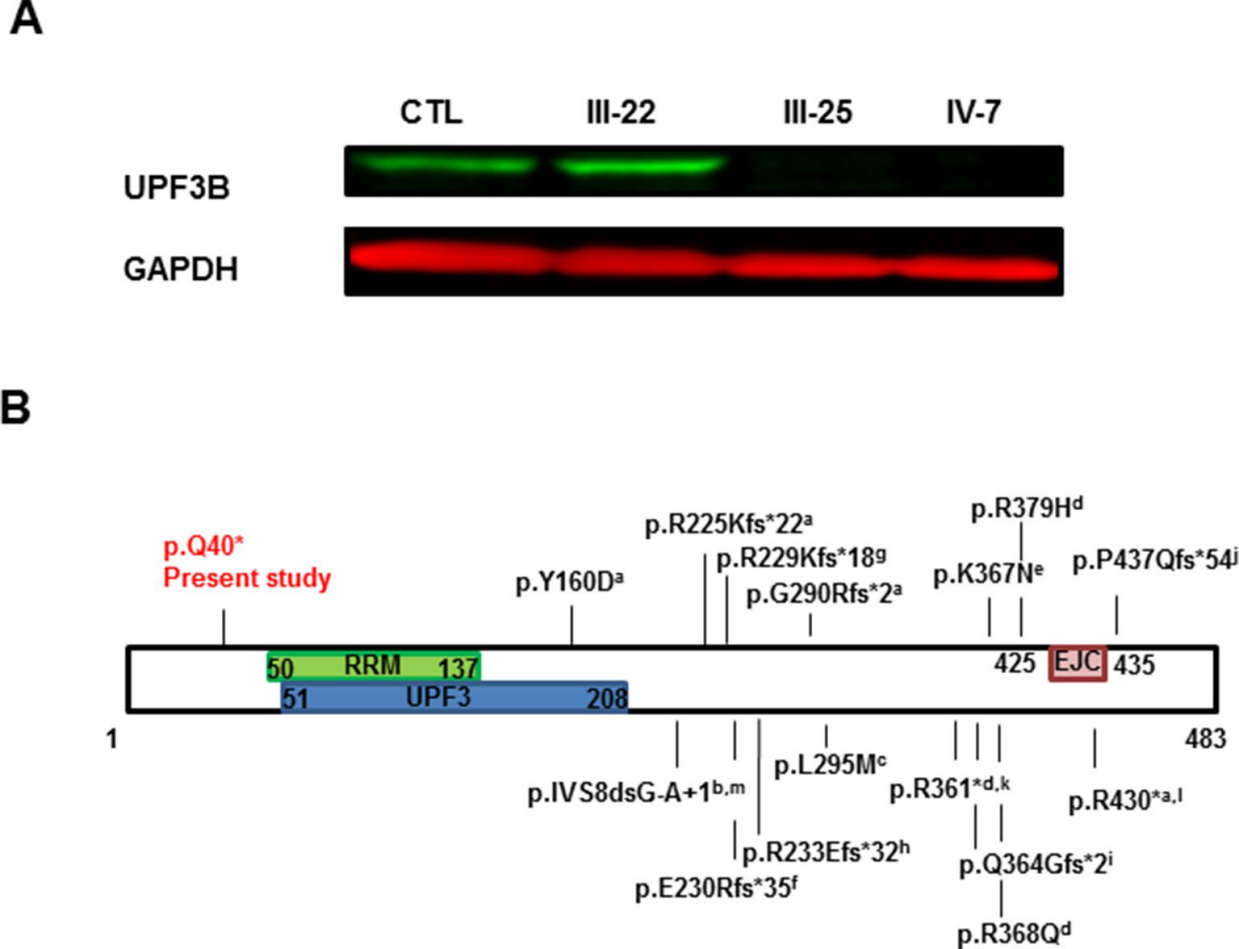
UPF3B protein analysis. **(A)** Western Blot analysis of PBMC samples obtained from peripheral blood of Control (CTL), carrier mother (III-22), affected uncle (III-25) and proband (IV-7). Both affected males do not have UPF3B protein expression. **(B)** Graph representation of UPF3B protein. Functional domains are represented, RRM: RNA recognition motif, UPF3: UPF3 domain and EJC: exon junction complex or binding domain. Known variants in UPF3B are also annotated: a. Tarpey et al. (2007); b. Chérot et al. (2018); c. Zhang et al. (2015); d. Laumonnier et al. (2010); e. Tzschach et al. (2015); f. Yavarna et al. (2015); g. Addington et al. (2011); h. Lynch et al. (2012); i. Soden et al. (2014); j. Hu et al. (2016);k. Xiong et al. (2015); l. Xu et al. (2013); m. Piton et al. (2013).

UPF3B protein expression was analyzed by Western Blot in PBMCs obtained from peripheral blood of 4 subjects: one nonrelated healthy control, III-22 (carrier), III-25 (affected male) and IV-7 (the index case, affected male). Our antibody used (A303-688A-T) recognizing the region between residue 300 and 350 of UPF3B protein demonstrated that there is no protein at least at these residues and therefore downstream of the 40 residue because of the stop variant in exon 1 (**Figure 3A**). The carrier female expresses the protein at levels similar to control as expected by complete skewed X inactivation (0:100).

## Discussion

In this report, we present a novel nonsense variant (c.118C > T; p.Gln40*) in the *UPF3B* gene identified in a male patient with ID and autism by NGS. The variant was also present in the other two living affected males and in seven carrier females, and it was absent from the available unaffected males. Therefore, it completely co-segregated with the disease within the family (**Figure 1A**). In addition, this variant is pathogenic because it introduces a premature termination codon (PTC) in the first exon of the gene and no UPF3B protein was found in samples from two of the patients (**Figure 3A**). Truncating variants seem to be frequent in *UPF3B* since 13 truncating variants, either nonsense (2), frameshift (10) or splicing variants (1), have already been reported in this gene in the HGMD.

The *UPF3B* gene (MIM 300298) is located in Xq24, and encodes a protein involved in nonsense-mediated mRNA decay. Variants in this gene were first described in four families with syndromic (Lujan–Fryns and FG like presentations), and non-syndromic X-linked intellectual disability and autism (Tarpey et al., 2007). Since then, more and more patients with ID have been studied by NGS and more variants have been reported, even deletions found by microarrays (Lovrecic et al., 2018). Although 21 variants (17 pathogenic) have been identified in *UPF3B* according to the HGMD, the spectrum of clinical features of patients with *UPF3B* variants remains insufficiently defined. So far, a small number of patients and families have been reported with detailed description of their phenotype (Tarpey et al., 2007; Tarpey et al., 2009; Laumonnier et al., 2010; Addington et al., 2011; Lynch et al., 2012; Xu et al., 2013) and only two of them have photographs (Tarpey et al., 2007; Lynch et al., 2012). We were able to obtain photographs of the five affected patients in childhood and in adulthood (**Figure 1B**). To our knowledge this is the first report showing the natural history of patients with *UPF3B* mutation.

The comparison between our cases and those reported previously is given in **Table 1**. As it has been described, there is remarkable clinical variability among the patients, even among those sharing the same variant, as is the case in the patients from our family. This wide variability between and within families has been previously described (Tarpey et al., 2007; Lynch et al., 2012). None of our patients have renal dysplasia (Lynch et al., 2012) or schizophrenia (Addington et al., 2011) but they all have some dysmorphic features while the families reported by Addington et al.(2011), Xu et al. (2013) and one of the first report by Tarpey et al. (2007) did not. So, the present family adds to the growing evidence of the clinical and genetic overlap in neurodevelopmental disorders. To highlight, physical and neurological phenotypes do not go together: III-10 and III-25 seem physically very similar (**Figure 1B**), but III-10 has a profound ID (IQ = 20.5) with autism and absent speech while III-25 has an IQ of 67, communicates well and is almost autonomous, working in an occupational centre for people with disabilities.

Another interesting patient is III-21 (**Figure 1**). Unfortunately we couldn't study him because he died many years ago; but it is tempting to speculate that this patient carried the *UPF3B* c.118C > T variant both from the pedigree analysis and because he presented with marfanoid-like features (slender figure, tall stature and long thin hyperextensible fingers and toes) which have been reported in patients with *UPF3B* variants, previously diagnosed as LF syndrome (OMIM 309520) (Tarpey et al., 2007). Another syndrome or a submicroscopic CNV (Callier et al., 2013), could have also been suspected. However we will never know if this patient had chromosomal imbalance or a different "*de novo*" mutation. In any case, his mother's DNA was studied with an X-linked panel finding only the *UPF3B* mutation of the family. Anyway, a study of 100 patients with marfanoid syndromes and ID did not find any pathogenic variant in *UPF3B*, suggesting that marfanoid habitus could be a relative non-specific feature of patients with ID (Callier et al., 2013). In our family, this is the only case with this phenotype providing further evidence for the wide clinical spectrum produced by the absence of UPF3B protein.

The UPF3B protein is an important component of the nonsense-mediated mRNA decay surveillance machinery and it has been proposed that it may have a potential function in the regulation of the expression and degradation of various mRNAs present at the synapse (Laumonnier et al., 2010) and hence this may explain the phenotypic variability. Despite this hypothesis, the mechanism by which PTC variants of *UPF3B* lead to ID is still unknown. In this sense, mRNA sequence of *UPF3B* with the p.Gln40* variant should be degraded by NMD, but it seems that it is not the case: *UPF3B* cDNA from blood samples was amplified in the proband and carrier mother (**Figure 2**) showing that the mRNA containing the PTC variant in the proband is not completely degraded by NMD. With these results, we could propose that the lack of UPF3B protein abrogates NMD mechanism, but some NMD has been reported for other nonsense variants in *UPF3B* (Tarpey et al., 2007) previously. In this sense, it has been proposed that NMD function can be partially rescued through UPF3A, a protein paralog of UPF3B (Nguyen et al., 2012, Alrahbeni et al., 2015)). On the other hand, there must be other genetic and non-genetic factors contributing to the variability in clinical expression (Lynch et al., 2012).

In any case, western blot analysis demonstrated that the c.118C > T variant leads to an absence of the complete UPF3B protein in blood in two of our affected individuals (**Figure 3A**). Even if there was some translation of the corresponding mRNA, all the functional domains would be missing because the nonsense variant is located before the three functional domains of the UPF3B protein: a RNA recognition motif (residues 50-137), a UPF3 motif (residues 51 to 208) and an EJC motif or exon junction complex binding domain (residues 425-435) (PFAM^3^, SMART^4^ databases), while the rest of the described variants in the UPF3B protein are located after some of these domains (**Figure 3B**). Therefore, although Tarpey et al. (2007) showed that the lack of the EJC is enough to the loss-of-function mutations of *UPF3B*, in our family the variant found would be much more likely to lead a complete lack of UPF3B function which is critical for neuronal differentiation (Alrahbeni et al., 2015) and explains the pathogenicity of the variant reported here.

## Concluding Remarks

In conclusion, the present study is the first report of a premature termination codon before the three functional domains of UPF3B protein, and cDNA and protein studies have demonstrated the absence of the complete UPF3B protein, implicating this variant directly with XLID, autism and some dysmorphic features.

## Data Availability Statement

The sequencing data generated and analyzed in this study (c.118C > T; p.Gln40* in the UPF3B gene NG_009241.1) can be found in the GenBank repository (https://www.ncbi.nlm.nih.gov/Genbank ) with the accession number for this nucleotide sequence: BankIt2260137 Seq1 MN447415.

## Ethics Statement

The studies involving human participants were reviewed and approved by Research ethics committee of Cruces University Hospital, Barakaldo, Spain. Written informed consent to participate in this study was provided by the participants' legal guardian/next of kin. Written informed consent was obtained from the individual(s), and minor(s)' legal guardian/next of kin, for the publication of any potentially identifiable images or data included in this article.

## Author Contributors

MIT provided patient clinical data and samples and designed the study; OV, NI, ABH, CM-B, EB, FM, MF, BS, and FB performed all the molecular studies; MIT and OV wrote the manuscript and all authors revised it critically, approved the final manuscript as submitted and agreed to be accountable for all aspects of the work.

## Funding

This work was funded by grant No. 2017111017, from the Health Department of the Government of the Basque Country.

## Conflict of Interest

The authors declare that the research was conducted in the absence of any commercial or financial relationships that could be construed as a potential conflict of interest.

## Acknowledgments

The authors would like to thank the family for their cooperation, for giving us photographs and for consenting to the publication of this manuscript. We also would like to thank Charles Schwartz for his suggestions and critical review of the manuscript and Cindy Skinner for sample coordination, both from Greenwood Genetic Center.

## Footnotes

1 Human Gene Mutation Database (HGMD) Professional 2019.1. URL: https://portal.biobase-international.com/hgmd/pro/start.php. Last accessed the 12th of June 2019.

2 ClinVar. URL: https://www.ncbi.nlm.nih.gov/clinvar/?term=UPF3B[gene]. Last accessed 23 September 2019.

3 PFAM Database. URL: https://pfam.xfam.org/.

4 Simple Modular Architecture Research Tool (SMART). URL: http://smart.embl-heidelberg.de/.
